# Immune suppressive signaling regulated by latent transforming growth factor beta binding protein 1 promotes metastasis in cervical cancer

**DOI:** 10.1590/1414-431X2022e12206

**Published:** 2023-01-09

**Authors:** Haiyan Gu, Wei Wang, Changdong Sun, Li Ding, Li Li, Peng Shu, Jun Xu

**Affiliations:** 1 Department Gynecology, The People's Hospital of Beilun District, Beilun Branch Hospital, The First Affiliated Hospital, Medical School, Zhejiang University, Beilun District, Ningbo, China; 2 Biomedical Big Data Center, Huzhou Maternity & Child Health Care Hospital, Huzhou, China; 3 Clinical Laboratory, The People's Hospital of Beilun District, Beilun Branch Hospital, The First Affiliated Hospital, Medical School, Zhejiang University, Beilun District, Ningbo, China; 4 Clinical Laboratory, Ningbo First Hospital, Ningbo, Zhejiang, China

**Keywords:** LTBP1, Cervical cancer, MDSC, Drug screening

## Abstract

Although metastasis is the major cause of death in cervical cancer, the mechanism of metastasis is still unclear. The mRNA expression and protein level of latent transforming growth factor beta binding protein 1 (LTBP1) were detected in tumor tissues and paracancerous tissues from in-house samples. Cell proliferation, cell cycle, migration, and *in vivo* metastasis were determined after LTBP1 was knocked down. Then, 13 drugs were screened, and the changes in cell apoptosis and proliferation and tumor metastasis were detected after drug treatment in shRNA cells. In our in-house samples, LTBP1 was lowly expressed in cervical cancer tissues. After LTBP1 knockdown, cell proliferation was increased, and the ability of *in vitro* migration and *in vivo* metastasis was enhanced. At the same time, the proportion of myeloid derived suppressor cells (MDSC) *in situ* increased, the proportion of T cells decreased, and transforming growth factor beta-1 (TGFβ1) signaling was activated. After carboplatin treatment, LTBP1 shRNA cell line apoptosis increased, metastasis *in vivo* was limited, and the proportion of MDSC *in situ* decreased. LTBP1 was lowly expressed in cervical cancer, and the inhibition of LTBP1 can improve the malignant degree of the tumor, and this process can be blocked by carboplatin.

## Introduction

Cervical cancer is one of the most common gynecological malignancies; there are 520,000 new cases and 270,000 related deaths reported annually (1). Metastasis is a major contributing factor to death in patients with cervical cancer, and once a patient has a metastasis event, the 5-year survival rate severely drops to 10% (2), making identification of the metastasis driver gene critical. Previous studies on tumors focused mainly on the molecular biological mechanisms of tumor cells, such as gene mutation, proliferation, and apoptosis signal pathway changes. In recent years, an increasing number of studies have found that the occurrence, development, metastasis, and prognosis of malignant tumors are closely related to the surrounding environmental factors of tumor cells (3). Tumor microenvironment (TME) is a comprehensive system induced by tumor cells to produce a large number of growth factors, chemokines, cytokines, and matrix metalloproteinases (MMPs). The cellular component of the TME includes tumor, stromal, and immune cells (4). The TME is characterized by hypoxia, acidosis, and interstitial hypertension, which play an important role in the occurrence, development, and metastasis of tumors (5). However, how the tumor cell induces the formation of the TME and the pre-metastasis niche remains unclear.

Latent transforming growth factor beta binding protein (LTBP) is a group of glycoproteins with a molecular weight of 120-220 kDa and contains calcium binding epidermal growth factor domain and 8-cys domain (6). LTBP can be divided into four types. The main functions of LTBP are related to the regulation of fibrinogen and tumor growth factor (TGF)-β1 (7). In our previous studies, we confirmed that the deletion of LTBP4 in liver cancer cells could induce the secretion of TGF-β1 (8). Similar to LTBP4, the function of LTBP1 is also closely related to the secretion of TGF-β1 (9). In most studies, *LTBP1* is increasingly being considered to be an oncogene, and expression of *LTBP1* is closely related to the activity of the Wnt signaling pathway and lung and bone metastasis in breast cancer (10). The overexpression of *LTBP1* in ovarian cancer results in distribution of TGF-β1 in the stromal tissue surrounding carcinoma cells (11), and in colorectal cancer, protein tyrosine phosphatases (PTPS) facilitate iNOS-mediated LTBP1 S-nitrosylation and LTBP1 protein degradation, thereby promoting the development of early colorectal cancer (12). However, the association between LTBP1 and cervical cancer has not been reported.

## Material and Methods

### Patient samples and cell culture

Ten pairs of cervical cancer and paracancerous tissue and 24 tumor tissue samples from patients with cervical cancer that received single treatment of carboplatin were obtained from Ningbo Beilun People's Hospital, China. Patients with tumor disappearance (last detected more than 4 weeks prior) were identified as carboplatin-response patients (complete response), and patients with partial response, progressive disease, and stable disease were identified as carboplatin-resistance patients. Cancer cell lines HELA, HT-3, SW756, MS751, and CAL39 were purchased from American Type Culture Collection (ATCC, USA). The 293t cells were kept in our laboratory, and all the above cell lines were cultured in DMEM with 10% FBS medium.

### q-RT-PCR

Gene expression at the mRNA level was analyzed by real-time quantitative reverse transcription PCR (qRT-PCR). Firstly, tissues or cells were lysed with TRI-ZOL reagent and then centrifuged at 8,000 *g* and 4°C for 15 min to remove the cell components. The supernatant was removed, chloroform was added, and the mixture was centrifuged at 8,000 *g* and 4°C for 15 min. mRNA was then precipitated by isopropanol and washed with ethanol. Finally, mRNA was quantified by measuring absorbance at 260/280 nm. Subsequently, mRNA was reverse transcribed using PrimeScript RT Reagent Kit with gDNA Eraser (RR047a, Takara Company, China), in accordance with the manufacturer’s protocol. Gene expression in the cDNA was then detected using TB Green Premix Ex Taq II (Tli RNase H Plus, RR820L, Takara Company,). Primer sequences were as follows: *LTBP1*: F 5′-TGAATGCCAGCACCGTCATCTC-3′, R 5′-CTGGCAAACACTCTTGTCCTCC-3′; *COL1A2:* F 5′-CTGGTGCTAAAGGAGAAAGAGG-3′, R 5′-ATCACCACGACTTCCAGCAGGA-3′; *COL3A1*: F 5′-TGGTCTGCAAGGAATGCCTGGA-3′, R 5′-CTTTCCCTGGGACACCATCAG-3′; *COL5A2:* F 5′-CAGGCTCCATAGGAATCAGAGG-3′, R 5′-CCAGCATTTCCTGCTTCTCCAG-3′; *TGF-βR2*: F 5′-GTCTGTGGATGACCTGGCTAAC-3′, R 5′-GACATCGGTCTGCTTGAAGGAC-3′; *TGF-β1*: F 5′-TACCTGAACCCGTGTTGCTCTC-3′, R 5′-GTTGCTGAGGTATCGCCAGGAA-3′; *SMAD7*: F 5′-TGTCCAGATGCTGTGCCTTCCT-3′, R 5′-CTCGTCTTCTCCTCCCAGTATG-3′; *TGF-βR3*: F 5′-TGGAGTCTCCTCTGAATGGCTG-3′, R 5′-CCATTATCACCTGACTCCAGATC-3′; *MAGI2:* F 5′-CACCGCTATGTCATCGACCTCA-3′, R 5′-CACCGCTATGTCATCGACCTCA-3′; *ZEB2*: F 5′-AATGCACAGAGTGTGGCAAGGC-3′, R 5′-CTGCTGATGTGCGAACTGTAGG-3′; *ANKRD1*: F 5′-CGACTCCTGATTATGTATGGCGC-3′, R 5′-GCTTTGGTTCCATTCTGCCAGTG-3′; *GAPDH*: F 5′-GTCTCCTCTGACTTCAACAGCG-3′, R 5′-ACCACCCTGTTGCTGTAGCCAA-3′. After detecting the expression of genes, expression of *LTBP1* was confirmed by the 2^-△△CT^ value.

### Western blot

Gene expression at the protein level was detected by western blot. Tissue or cells were lysed by RIPA buffer on ice for 30 min; then, loading buffer was added and samples were boiled for 15 min. The protein in the sample was separated on a 12% SDS-PAGE (sodium dodecyl-sulfate polyacrylamide gel electrophoresis) gel and transferred to a PVDF (polyvinylidene difluoride) membrane. The membrane was blocked with 5% BSA (bovine serum albumin) and labeled with the corresponding primary antibody. Primary antibodies used in this experiment included ZEB2 (Zinc finger E-box-binding homeobox 2) (ab138222), SMAD7 (ab90086), p-SMAD3 (ab52903), TGF-β1 (ab92486), LTBP1 (ab78294), and GAPDH (glyceraldehyde-3-phosphate dehydrogenase) (ab8245), all of which were purchased from Abcam (USA). The membrane was then labeled with a secondary antibody; all the secondary antibodies were purchased from ZhongShan Golden Bridge Biotechnology (China). The gene expression at the protein level was determined by a chemiluminescence system (Pierce Biotechnology, USA).

### shRNA transduction and lenti-virus package

LTBP1 shRNA vector were all purchased from Sigma-Aldrich Co. (USA). For the *in vitro* experiments, cells were divided with CON and LTBP1-shRNA, then transfected with scramble and LTBP1-shRNA vector. The transfection of vector was performed using Lipofectamine 2000 reagent (Thermo Fisher Scientific, USA). For the *in vivo* experiments, scramble or LTBP1-shRNA vector was mixed with PMD2.G and pSPAX2 vector, ratio of 4:1:3, then the mixture was transfected in 293t cell lines. The supernatant, which contained the virus, was collected and finally cells were infected with scramble (CON group) and LTBP1-shRNA (LTBP1-shRNA group) for 48 h. Multiplicity of infection (MOI) was 10.

### Cell apoptosis, proliferation, and wound healing assay

Cell apoptosis in this experiment was determined using Annexin V APC/PI (propidium iodide) double staining kit (BD Biosciences, USA). First, cells were harvested by trypsin and washed three times with phosphate-buffered saline (PBS). Cells were then suspended in binding buffer, and 5 µL of Annexin V APC antibody was added. After culturing on ice for 15 min, 5 µL of PI was added and the percentage of Annexin V to PI-positive cells was detected using a FACS (fluorescence-activated cell sorting) Calibur machine (Becton, Dickinson and Company, USA). Cell proliferation was determined with a CCK8 (Cell Counting Kit 8) kit (Dojindo Molecular Technologies, USA). Cells (1,000) were seeded onto a 96-well plate and 1/10 volume of CCK8 was added. After culturing at 5% CO_2_ and 37°C for 2 h, cells were scanned using a Varioskan Flash multimode microplate reader (Thermo Fisher Scientific, USA) to detect the absorbance at 450 nm, and the proliferation index was calculated as: OD450 nm at time-point/OD 450 nm at pre-culturing. A wound healing assay was used to detect cell migration. First, cells were seeded onto a 24-well plate, cultured at 5% CO_2_ and 37°C, and then the culture surface was scratched with sterile 10 µL pipette tips. Three positions were selected and, finally, an image was captured at 0 and 12 h.

### Cell cycle

The cell cycle in this study was detected by staining cells with a high PI concentration (50 mg/mL). Cells were first harvested with trypsin, washed three times with PBS, and fixed with 70% ethanol overnight. PI was then added to the cells and cells were cultured for 20 min at 20°C. Cell cycle was detected using a FACS Calibur machine.

### *In silico* analysis

All *in silico* analyses performed in this study used the R 4.0.5 software (http://www.r-project.org/). Survival analysis: mRNA expression profile and clinical information of TCGA (The Cancer Genome Atlas) were downloaded from the BROAD Institute website (https://gdac.broadinstitute.org/); the name of the gene expression file is ‘CESC.rnaseqv2__illuminahiseq_rnaseqv2__unc_edu__Level_3__RSEM_genes_normalized__data.data', and the clinical information file is ‘CESC.merged_only_omf_clin_format'. Gene expression of patients was normalized by log2 transformation, and the mean of LTBP1 expression was calculated. Furthermore, patients with an LTBP1 expression level lower than the mean were grouped in the LTBP1 low group and those with levels higher than the mean were grouped in the LTBP1 high group. The relationship between the expression of LTBP1 and overall survival rate of patients was determined by univariate COX analysis. Gene Ontology (GO) annotation: gene expression data of the GSE9750 dataset was downloaded using the GEOquery package, annotated with the GPL96 platform, and the data was normalized by log2 transformation. The genes that highly correlated with LTBP1 expression were screened using the criteria of abs (r^2^)>0.5 and P<0.05. Finally, pathway enrichment of the highly correlated genes was determined using the clusterProfiler package (https://www.rdocumentation.org/packages/clusterProfiler).

### Drug screening

Thirteen drugs that included bevacizumab (A2006), sunitinib (S7781), pazopanib (S3012), lapatinib (S2111), cediranib (S1017), cetuximab (A2000), trastuzumab (A2007), gefitinib (S1025), erlotinib (S7786), olaparib (S1060), topotecan (S9321), cisplatin (S1166), and carboplatin (S1215) were purchased from Selleck Chemicals LLC (USA). The half maximal inhibitory concentration (IC50) of the drugs was obtained from the Selleck Chemicals LLC and then, HELA and HT-3 cells were treated with IC50 of the drugs for 48 h. mRNA from cells treated with or without the drugs was extracted and the expression of *LTBP1* at the mRNA level was detected.

### CyTOF and FACS

In the CyTOF (cytometry by time of flight) experiment, single cells from the primary tumors were obtained by digesting them with type I collagen for 2 h. Cells were filtered with a 40 µM strainer and counted. Three million cells were labeled with CD3E, CD8A, CD11B, LY6G, and LY6C antibodies using Fluidigm mass cytometry labeling according to the manufacturer's instructions (Fluidigm, USA). Finally, the .fcs files of CyTOF experiment were read using the flowCore package (https://bioconductor.org) in R language after normalization. Subsequently, cells were clustered with the Rtsne package (https://cran.r-project.org), and the result was visualized using ggplot2 package. In the FACS experiment, primary tumor cells were extracted and digested with type I collagen. Solutions containing single cells were labeled with CD11B, LY6G, and LY6C antibodies, and the percentage of these labeled cells was calculated using the FACS Calibur machine.

### Nude mice experiment

Nude mice used in this study were purchased from Beijing Wei-tong Li-hua Laboratory Animals and Technology (China). All experiments complied with the guidelines of the National Institutes of Health guide for the Care and Use of Laboratory Animals (NIH Publications No. 8023, revised 1978, USA). Furthermore, all experiments involving mice were approved by the Ethics Committee of Ningbo Beilun People's Hospital [approval reference number: 2021-73 (YS)].

#### In vivo metastasis experiment

Ten nude mice were randomly divided into CON and LTBP1 shRNA groups. Mice were injected with 1×10^6^ cells through the tail, and after 2 weeks, they were sacrificed and dissected. Using a fluorescence microscope, the GFP (green fluorescent protein) signal was detected in lungs, and the signal was quantified by ImageJ 1.52K software (NIH).

#### Subcutaneous tumor bearing experiment

Ten Balb/c mice were divided into CON and LTBP1 shRNA groups. Mice were subcutaneously injected with 1×10^7^ tumor cells. Two weeks after injection, the primary tumors were collected and digested to produce single cells. The profile of the immune cells in the primary tumors were detected using CyTOF and FACS.

#### Drug treatment

In the drug treatment experiment, mice were injected with 1×10^7^ tumor cells subcutaneously or through the tail vein. One week after injection, the mice were intraperitoneally injected with carboplatin (10 mg/kg), twice a week for 2 weeks.

## Results

### Consistent low expression of LTBP1 in cervical carcinoma tissue

**Figure 1:**
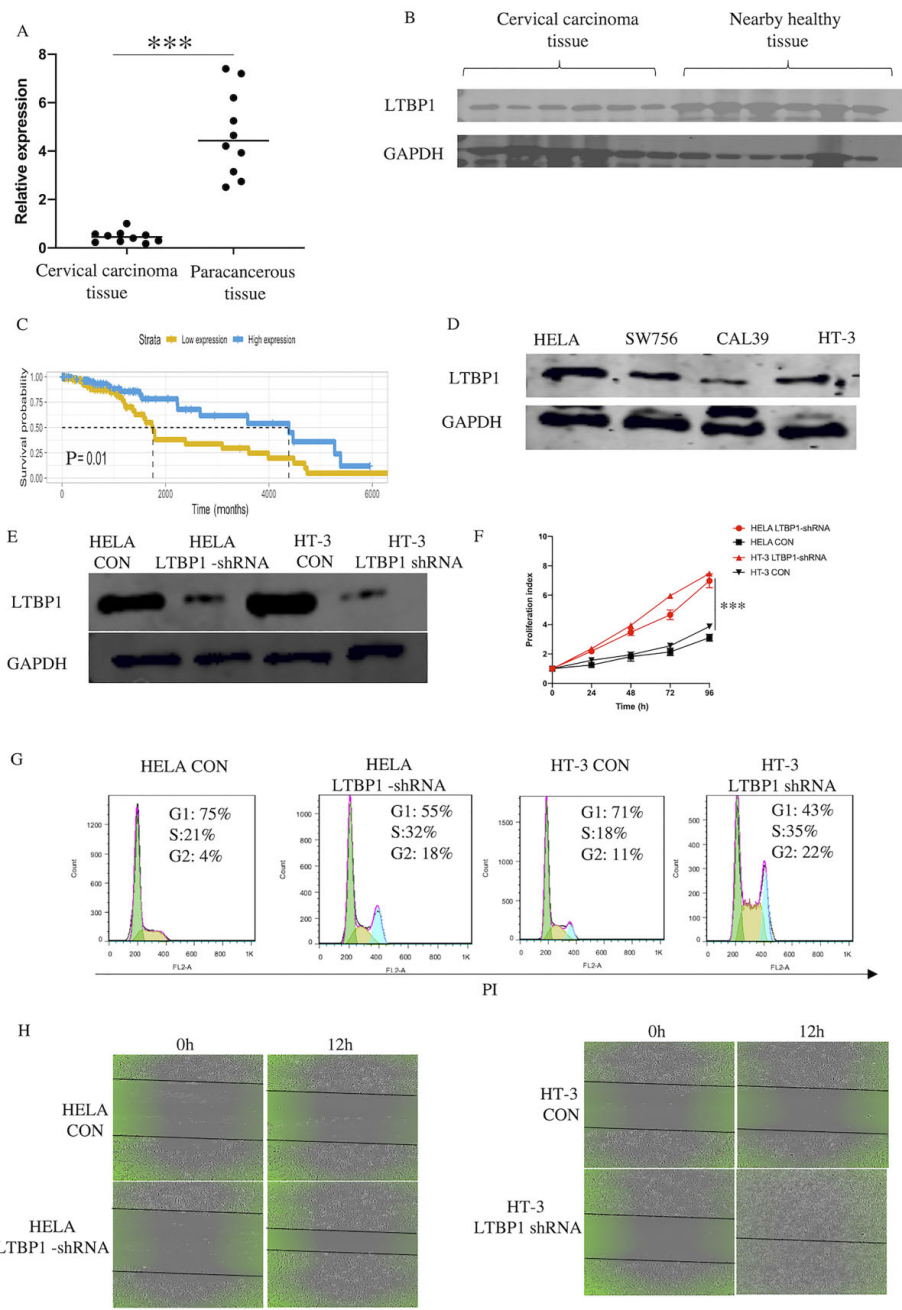
Consistently low expression of LTBP1 increases proliferation and migration of cervical cancer cells. **A**, Expression of LTBP1 in 10 paired cervical carcinoma and paracancerous tissues by qPCR. **B**, LTBP1 protein level in 7 pairs by western blot of tissue chosen from panel **A**. **C**, Relationship between overall survival rate and expression of LTBP1 from TCGA database, using univariate Cox analysis. **D**, Expression of LTBP1 in 4 cervical cancer cell lines was detected by western blot. **E**, HELA and HT-3 were transfected with CON and LTBP1-shRNA vector for 48 h, using western blot to detect the change of LTBP1 protein expression. **F**, Cell proliferation using CCK8 staining. **G**, Change of cell cycle using PI staining. **H**, Change of cell migration in HELA CON, HELA LTBP1-shRNA, HT-3 CON, and HT-3 LTBP1-shRNA by wound healing assay. Data are reported as means and SD. ***P<0.001 (*t*-test and ANOVA).

In this study, to determine the role of LTBP1 in cervical cancer, we examined the relative expression of *LTBP1* in 10 pairs of cervical carcinoma and paracancerous tissues, and it was found to be substantially lower in cervical carcinoma tissues (1.23±0.34) compared with that in paracancerous tissues (4.32±2.17) (Figure 1A). We further validated this pattern at the protein level. As the size of three of the tissue pairs was limited, we detected the expression of LTBP1 in the other seven pairs. Similar to mRNA level, the LTBP1 protein level was low in cervical carcinoma tissues (Figure 1B). In the TCGA database, patients that had a lower expression of *LTBP1* had a shorter overall survival rate (Figure 1C). Together, these results led us to conclude that LTBP1 has a reduced expression in cancer tissue.

We then investigated the expression of LTBP1 in cervical cancer cell lines and found that HELA and HT-3 had a relatively higher LTBP1 protein level compared with 2 other cell lines (Figure 1D). Thus, we chose the HELA and HT-3 cell lines for further experiments. First, they were transfected with CON or *LTBP1* shRNA vector for 48 h, and the change of LTBP1 protein level was detected (Figure 1E). We then confirmed that the absence of *LTBP1* promotes proliferation (Figure 1F) and activates the cell cycle (Figure 1G). As tumor metastasis accounts for the majority of cancer-related deaths in cervical patients, we also detected the change in cell migration. Results showed that cell migration was highly elevated in HELA and HT-3 cells after LTBP1 was inhibited (Figure 1H). These results revealed that low expression of LTBP1 in cancer cells could increase their proliferation and possibly invasion.

### Low expression of LTBP1 increased aggressiveness of cervical carcinoma

**Figure 2:**
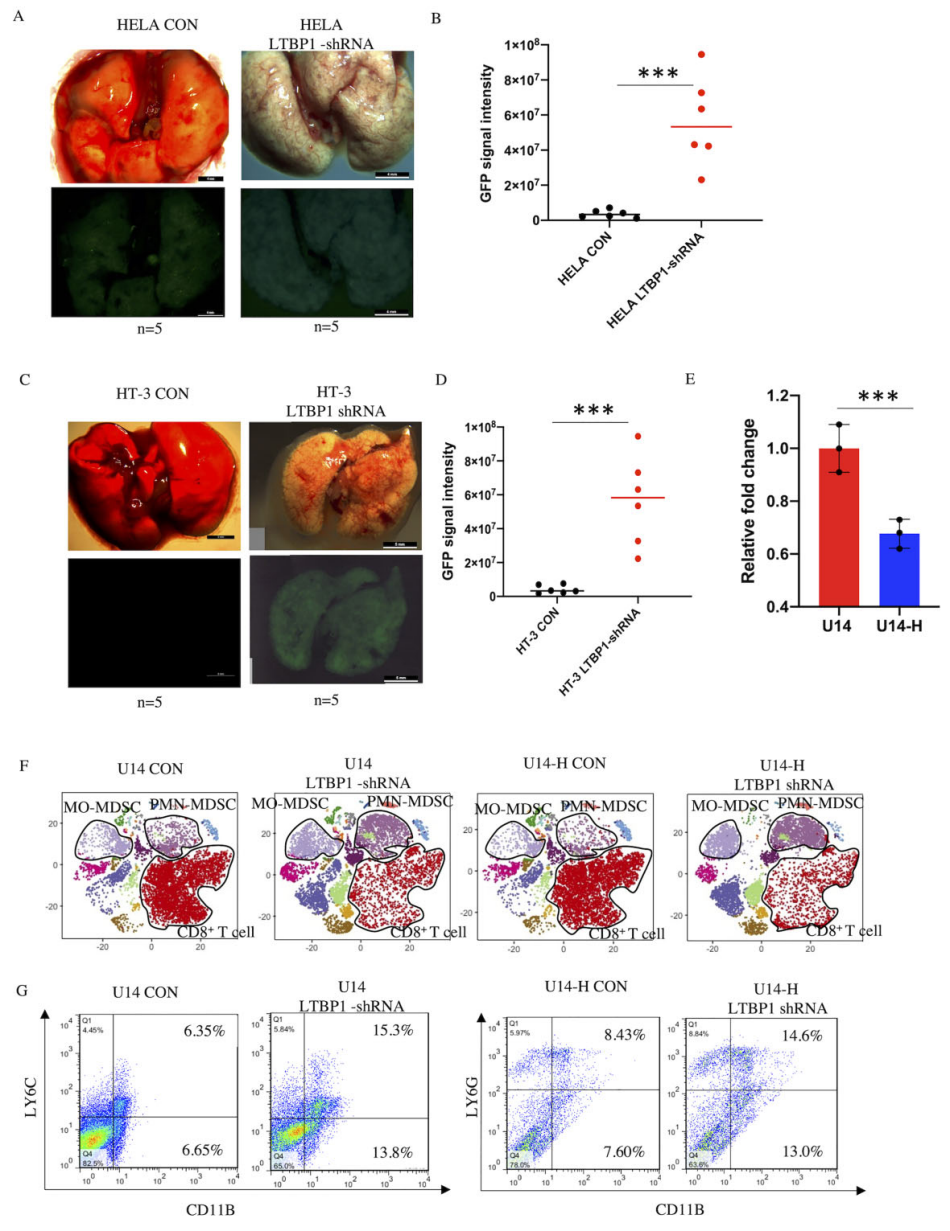
Inhibition of LTBP1 promoted metastasis incidence and immune suppression signal. **A**, Representative images of lungs after 2 weeks of tail vein injection with parental HELA cells (CON) (n=6 mice) and HELA LTBP1 shRNA cells (HELA-LTBP1 shRNA) (n=6 mice per group, scale bar: 4 mm). **B**, Quantification of the green fluorescent protein (GFP) intensity of lung shown in the lower panels of **A**. **C**, Representative images of lungs after 2 weeks of tail vein injection with parental HT-3 cell (CON) (n=6 mice per group) and HT-3 LTBP1 shRNA cell (HT-3 LTBP1 shRNA) (n=6 mice per group, scale bar: 5 mm). **D**, Quantification of GFP intensity of lung shown in lower panels of **C**. **E**, Expression of LTBP1 in U14 and U14-H cell lines in mRNA level. Data are reported as means and SD. **F**, Profiles of immune cell populations from primary tumor implanted with U14 CON, U14 LTBP1-shRNA, U14-H CON and U14-H LTBP1-shRNA cells in Balb/c mice analyzed with antibodies against CD45, CD8, CD4, CD3, Ly6G, and Ly6C by cytometry by time of flight analysis (n=5 mice per group). **G**, Representative images of myeloid derived suppressor cells from primary tumor implanted with U14 CON, U14 LTBP1-shRNA, U14-H CON, and U14-H LTBP1-shRNA cells in nude mice and analyzed by FACS. ***P<0.001 (*t*-test).

Most mice injected with HELA CON cells had metastasis signals in the lungs, and in the *LTBP1* shRNA group, the GFP signal was increased due to the accumulation of tumor cells (Figure 2A and B). Similar to HELA cells *in vivo* results, the GFP signal was highly enhanced in the *LTBP1* shRNA group (Figure 2C and D), suggesting that low expression of *LTBP1* could increase the metastatic ability of cervical cancer cell lines. Based on previous research (8), we confirmed that LTBP4 could regulate the secretion and activation of TGF-β1 in various tissues. Furthermore, previous studies have suggested that the elevated levels of TGF-β1 could inhibit the proliferation of CD8 T cells and increase the percentage of myeloid derived suppressor cells (MDSCs), which could further generate immune suppression signal in the TME. To validate this theory, we chose the U14 cell line, as U14 is partially incompatible in Balb/C mice and only 50% of mice would likely develop a primary tumor post-injection. Therefore, we sorted single cells from the primary tumor in mice, which were subcutaneously injected with the U14 cell line. After culturing the cells from the primary tumor, we successfully increased the percentage of tumorigenesis to 90%, and determined that the expression of *LTBP1* was significantly lower in the U14-H group compared with that in the U14 group (Figure 2E), suggesting that the low expression of *LTBP1* could promote the TME modulation. We also performed CyTOF to clarify the profile of immune cells in the primary tumor. The results showed that, in the primary tumor derived from mice injected with U14-H cells, the percentage of CD8+ cells only slightly increased compared with that in the U14 CON group. We confirmed that monocytic myeloid-derived suppressor cells (MO-MDSCs), neutrophils, and polymorphonuclear myeloid-derived suppressor cells (PMN-MDSCs) increased in the *LTBP1* shRNA group compared with those in the CON group, and CD8+ T cell level dramatically decreased in the *LTBP1*-shRNA group (Figure 2F). Further validation by FACS analysis confirmed the elevation of MO-MDSCs and PMN-MDSCs (Figure 2G). These data demonstrated marked activation of immune suppression signaling by low expression of LTBP1 in tumor cells.

### Carboplatin can block LTBP1-regulated TGF-β1 pathway

**Figure 3:**
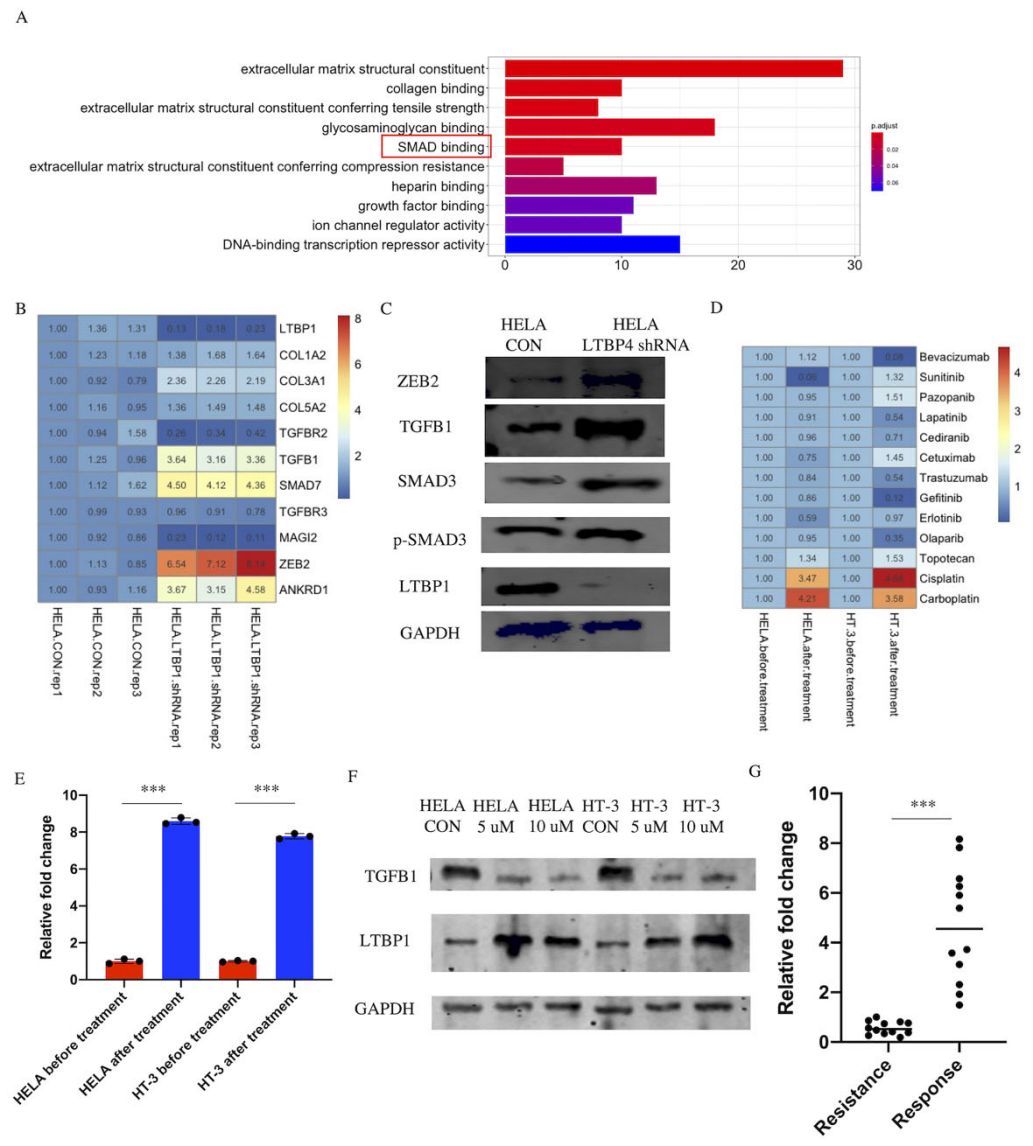
Identification of pathway and drugs related with LTBP1. **A**, Top ten activated pathways based on highly correlated genes of cervical cancer tissue from the GSE9750 dataset. **B**, Expression of SMAD binding genes with or without expression of LTBP1 shRNA in HELA cells at the mRNA level. **C**, Protein level of ZEB2, TGFB1, SMAD7, p-SMAD3, and LTBP1 in HELA cell with or without expression of LTBP1 shRNA. **D**, mRNA level of LTBP1 after treatment with 13 drugs. **E**, mRNA level of LTBP1 in HELA and HT-3 cells with or without treatment with 10 μM of carboplatin. Data are reported as means and SD. **F**, Protein level of TGFB1 and LTBP1 in HELA and HT-3 cells treated with different concentrations of carboplatin. **G**, LTBP1 gene expression in clinical resistance (n=12 patients) and response (n=12 patients) to a single treatment with carboplatin from in-house data. ***P<0.001 (*t*-test).

LTBP1 regulates the secretion of TGF-β1, but the exact mechanism of LTBP1-induced immune suppression signaling in cervical cancer remains unknown. Thus, we extracted gene expression profile from the GSE9750 dataset and identified 520 genes that were highly correlated with LTBP1 by using the criteria of abs (r^2^)>0.5 and P<0.05. The 520 genes were then annotated by GO. In the ‘Molecular Function’ enrichment, the pathway named ‘SMAD binding’ was highly enriched (Figure 3A), and 11 genes belonging to the enriched ‘SMAD binding’ pathway were detected in the HELA cells with or without expression of LTBP1 shRNA. We further confirmed that the TGF-β1 level was highly increased in LTBP1 inhibited cells. The *TGF-β1, SMAD7*, and downstream gene *ZEB2* were also increased at the mRNA and protein levels. Surprisingly, SMAD3 pathway remained unchanged after LTBP1 inhibition (Figure 3B and C). These results suggested that the TGF-β1-regulated LTBP1 specifically regulates the SMAD7 pathway.

Given its ability to increase metastasis and activate immune suppressive signaling, we attempted to block LTBP1-related metastasis. We treated the HELA and HT-3 cells with commonly used drugs in cervical cancer treatment. Both cisplatin and carboplatin could induce the expression of LTBP1 in mRNA level. The increased LTBP1 expression post-cell exposure might indicate the suppressive ability of this target. Topotecan slightly induced the LTBP1 expression; however, there was no stable pattern of regulation among other drugs (Figure 3D). Compared with cisplatin, carboplatin is a relatively new drug, which has less side effects and a broader spectrum. We validated the expression of LTBP1 in HELA and HT-3 cells, which were treated with carboplatin, and confirmed that LTBP1 was induced at the mRNA and protein levels. Moreover, expression of TGF-β1 was downregulated by carboplatin, suggesting that carboplatin could block the LTBP1-regulated pathway (Figure 3E and F). In the clinical samples, expression of LTBP1 was found to be increased in tumor tissue obtained from patients with cervical cancer that were resistant to carboplatin. Together, these results indicated that patients that responded to carboplatin had higher LTBP1 expression and that carboplatin could induce LTBP1 expression, suggesting that carboplatin might be able to block the increased proliferation and metastasis driven by the LTBP1^low^ state (Figure 3G).

### Restoration of immune suppressive signal by carboplatin treatment

**Figure 4:**
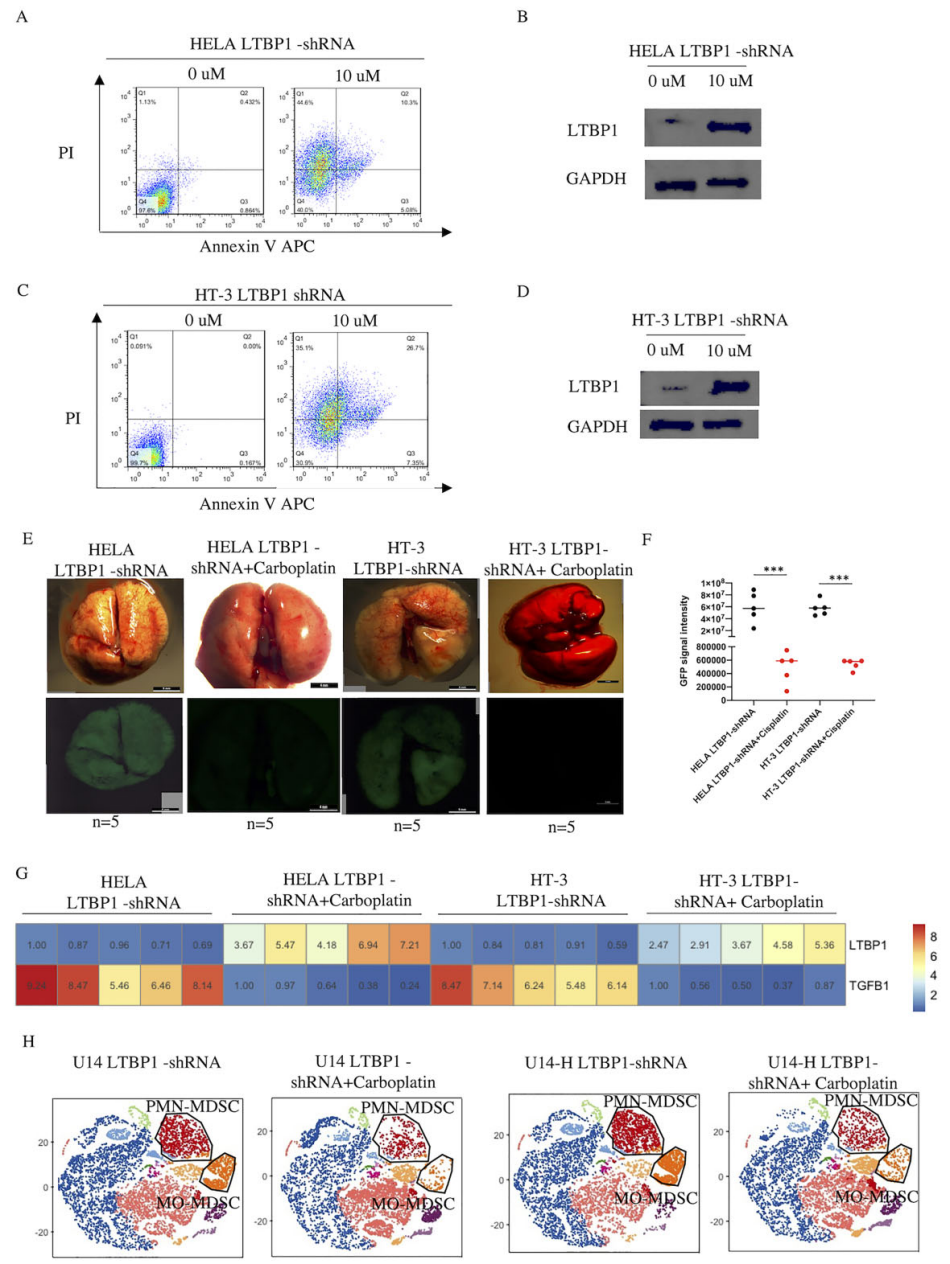
Activation of cytotoxic signal by carboplatin treatment. **A**, Cell apoptosis in HELA LTBP1 shRNA cells treated with 10 μM of carboplatin. **B**, Expression of LTBP1 in HELA LTBP1 shRNA cells treated or not with carboplatin. **C**, Cell apoptosis in HT-3 LTBP1 shRNA cells treated with 10 μM of carboplatin. **D**, Expression of LTBP1 in HT-3 LTBP1 shRNA cells treated or not with carboplatin. **E**, Nude mice were injected with HELA LTBP1 shRNA or HT-3 LTBP1 shRNA cells in the tail vein; after 1 week of injection, mice were treated or not with 10 mg/kg of carboplatin for 2 weeks (n=5 mice per group). **F**, Quantification of green fluorescent protein (GFP) intensity of lungs shown in lower panels of **E** (n=5 mice per group); ***P<0.001 (*t*-test). **G**, Expression of LTBP1 and TGFB1 in lungs from mice injected with HELA LTBP1 shRNA, HT-3 LTBP1 shRNA cells, and treated or not with carboplatin. **H**, Profiles of immune cell populations from primary tumor implanted with U14 LTBP1-shRNA, U14-H LTBP1-shRNA in Balb/c mice and treated or not with carboplatin, analyzed with antibodies against CD45, CD8, CD4, CD3, Ly6G, and Ly6C by cytometry by time of flight analysis (n=5 mice per group).

To further explore the effect of carboplatin, we treated HELA LTBP1 shRNA cells that had a higher proliferation index compared with HELA cells (Figure 1F). After 48 h of treatment, cell apoptosis (Figure 4A) and expression of LTBP1 was increased in HELA cells treated with carboplatin (Figure 4B), suggesting that carboplatin could block cell proliferation through the regulation of LTBP1 expression. A similar pattern was observed in HT-3 cells (Figure 4C and D). As tumor metastasis accounts for most cancer-related deaths and low expression of LTBP1 in tumor cells highly promotes metastasis *in vivo*, we injected HELA LTBP1 shRNA or HT-3 LTBP1 shRNA cells into the tail vein of nude mice, after which mice were intraperitoneally injected with carboplatin (10 mg/kg). After 2 weeks of treatment, GFP signal was significantly decreased (Figure 4E and F), and the expression of LTBP1 in the lungs was increased and that of TGF-β1 was decreased in mice treated with carboplatin (Figure 4G). These results indicated that LTBP1 is a protective factor in cervical cancer. Treatment with carboplatin not only arrested the LTBP1-related metastasis *in vivo* but also downregulated the TGF-β1 signaling in metastatic tumors, and the mechanism was found to be regulated by LTBP1 expression. Finally, using CyTOF, we showed that carboplatin decreased the percentage of immune suppressive PMN-MDSCs and MO-MDSCs (Figure 4H).

## Discussion

According to a WHO report, about 500,000 new cases of cervical cancer are reported globally every year, second only to breast cancer (13). Among them, 150,000 new cases occurred in China, accounting for about one-third of the world's gynecological malignancies (14). Therefore, it is important to study the pathogenic factors of cervical cancer and prevent its occurrence.

Although in most of the current studies, LTBP1 increasingly appeared as an oncogene (15), in our study, we confirmed that LTBP1 had a low expression in cervical cancer tissue samples compared with that in adjacent tumor tissue. The overall survival rate of patients with cervical cancer and expression of LTBP1 was significantly lower, which preliminarily confirmed that in cervical cancer, LTBP1 might be a tumor suppressor gene. Through further cell biology experiments, we confirmed that the inhibition of LTBP1 can promote the proliferation and metastasis of tumor cells, further indicating that *LTBP1* functions as a tumor suppressor gene in cervical cancer.

MDSC is a group of immature bone marrow cells, including immature dendritic cells (16), neutrophils (17), and macrophages (18). MDSCs can be divided into two subtypes: CD14+ monocytes and CD15+ granulocytes, which reflect the immune status of the body and are related to the occurrence and development of tumors (19). The number and proportion of MDSCs in the blood and tumor tissue of tumor bearing mice and peripheral blood and tumor tissue of patients with tumors were significantly increased (20) throughout the duration of tumor occurrence (21), and they were correlated with tumor size and degree of malignancy. In this study, the proportion of immune cells in tumors *in situ* was detected by the CyTOF test (22). The results showed that the level of PMN-MDSCs and MO-MDSCs increased significantly after LTBP1 expression was decreased, while the proportion of CD8+ T cells decreased, indicating that the immunosuppressive signaling pathway in the TME was activated after LTBP1 downregulation. Previous studies indicated that LTBP1 could regulate the secretion of TGF-β1 (9), and elevated TGF-β1 in the TME would activate immune suppression signaling. In our experiment, low expression of LTBP1 could increase the expression of *TGF-β1, SMAD7*, and downstream gene *ZEB2*, suggesting that LTBP1 could regulate the TGF-β1/SMAD7/ZEB2 axis.

To block the metastasis induced by lower LTBP1 expression, we used the method of drug screening, and confirmed carboplatin as a candidate drug. Through cell biology experiments and animal experiments, we showed that carboplatin could inhibit the proliferation of LTBP1 knockdown cell lines through the regulation of LTBP1 expression. At the same time, the increase of PMN-MDSCs and MO-MDSCs in the TME caused by LTBP1-knockdown cells was also blocked by carboplatin. The increase of tumor metastasis induced by LTBP1 knockdown can be arrested by carboplatin.

In conclusion, we confirmed the role of LTBP1 in cervical cancer and provided a reference for blocking the increase of tumor metastasis caused by LTBP1 deletion.
